# Congenital Hyperinsulinism in Humans and Insulin Secretory Dysfunction in Mice Caused by Biallelic *DNAJC3* Variants

**DOI:** 10.3390/ijms25021270

**Published:** 2024-01-20

**Authors:** Alena Welters, Oliver Nortmann, Laura Wörmeyer, Clemens Freiberg, Daniel Eberhard, Nadine Bachmann, Carsten Bergmann, Ertan Mayatepek, Thomas Meissner, Sebastian Kummer

**Affiliations:** 1 Department of General Pediatrics, Neonatology and Pediatric Cardiology, Medical Faculty and University Hospital Düsseldorf, Heinrich Heine University Düsseldorf, D-40225 Düsseldorf, Germany; olnor100@uni-duesseldorf.de (O.N.); mayatepek@med.uni-duesseldorf.de (E.M.); thomas.meissner@med.uni-duesseldorf.de (T.M.); sebastian.kummer@med.uni-duesseldorf.de (S.K.); 2 Institute of Metabolic Physiology, Heinrich Heine University Düsseldorf, D-40225 Düsseldorf, Germany; daniel.eberhard@uni-duesseldorf.de; 3 Department of Pediatrics and Adolescent Medicine, Pediatric Endocrinology, University Medicine Göttingen, D-37075 Göttingen, Germany; cfreib@med.uni-goettingen.de; 4 Medizinische Genetik Mainz, Limbach Genetics, D-55128 Mainz, Germany; nadine.bachmann@medgen-mainz.de (N.B.); carsten.bergmann@medgen-mainz.de (C.B.)

**Keywords:** hyperinsulinemic hypoglycemia, endoplasmic reticulum stress, calcium leak, intracellular calcium homeostasis, monogenic diabetes, BiP/GRP78 co-chaperone

## Abstract

The BiP co-chaperone DNAJC3 protects cells during ER stress. In mice, the deficiency of DNAJC3 leads to beta-cell apoptosis and the gradual onset of hyperglycemia. In humans, biallelic *DNAJC3* variants cause a multisystem disease, including early-onset diabetes mellitus. Recently, hyperinsulinemic hypoglycemia (HH) has been recognized as part of this syndrome. This report presents a case study of an individual with HH caused by *DNAJC3* variants and provides an overview of the metabolic phenotype of individuals with HH and *DNAJC3* variants. The study demonstrates that HH may be a primary symptom of DNAJC3 deficiency and can persist until adolescence. Additionally, glycemia and insulin release were analyzed in young DNACJ3 knockout (K.O.) mice, which are equivalent to human infants. In the youngest experimentally accessible age group of 4-week-old mice, the in vivo glycemic phenotype was already dominated by a reduced total insulin secretion capacity. However, on a cellular level, the degree of insulin release of DNAJC3 K.O. islets was higher during periods of increased synthetic activity (high-glucose stimulation). We propose that calcium leakage from the ER into the cytosol, due to disrupted DNAJC3-controlled gating of the Sec61 channel, is the most likely mechanism for HH. This is the first genetic mechanism explaining HH solely by the disruption of intracellular calcium homeostasis. Clinicians should screen for HH in DNAJC3 deficiency and consider *DNAJC3* variants in the differential diagnosis of congenital hyperinsulinism.

## 1. Introduction

Congenital hyperinsulinism (CHI) is a rare genetic disorder that causes recurrent episodes of hyperinsulinemic hypoglycemia. If left untreated, it can lead to long-term neurological sequelae [1,2]. The genetic and molecular mechanisms of CHI are diverse. They can directly affect the function of the pancreatic K_(ATP)_ channel and other channel/transporter proteins, such as L-type voltage-gated calcium channels. Alternatively, they can impact distinct metabolic pathways that converge on the beta-cell ATP content, leading to membrane depolarization via K_(ATP)_ channel closure, the influx of extracellular calcium, and ultimately, insulin release [3]. In addition, CHI has been reported as a component of various rare genetic syndromes, such as overgrowth syndromes, chromosomal and monogenic developmental syndromes, congenital disorders of glycosylation, and syndromic channelopathies [4].

Recently, it has been recognized that hyperinsulinemic hypoglycemia is part of a syndrome caused by biallelic loss-of-function mutations in the DnaJ Heat Shock Protein Family (Hsp40) Member C3 gene (*DNAJC3*, also known as *P58^IPK^*) [5,6]. Previously, biallelic *DNAJC3* variants have been linked to multisystem neurodegeneration with ataxia, peripheral neuropathy, cognitive impairment, sensorineural hearing loss, and retinal dystrophy as well as short stature, hypothyroidism, facial dysmorphism, mild skeletal bone deformities, and early-onset diabetes mellitus associated with pancreatic atrophy [7,8,9,10,11]. DNAJC3 acts as an endoplasmic reticulum (ER)-resident co-chaperone of the binding immunoglobulin protein BiP (GRP78). DNAJC3 plays multiple roles in protecting the cells from ER stress. As part of the unfolded protein response (UPR), it is upregulated and translocated into the ER lumen. There, it works alongside BiP to restore ER homeostasis by refolding misfolded proteins. During periods of sustained UPR signaling, DNAJC3 further assists in returning the ER to normal homeostasis by indirectly enhancing the initiation of protein translation. This is achieved through the inhibition of PKR-like endoplasmic reticulum kinase (PERK), which restores protein levels and supports cellular processes post-ER stress [12]. Conversely, the deficiency of DNAJC3 disrupts UPR signaling, triggers ER stress, and induces apoptosis. This has been demonstrated in INS-1E cells, primary rat beta cells, human islets, and retinal neurons [9,13,14]. In mice, knockout of *Dnajc3* causes beta-cell apoptosis, accompanied by hypoinsulinemia and the gradual onset of hyperglycemia as the mice mature [15,16]. However, an explanation for the bi-phasic phenotype of DNAJC3 deficiency in humans, with hyperinsulinemic hypoglycemia that remits in childhood/adolescence, eventually evolving into diabetes later in life, has not yet been found.

Here, we describe one individual with a biallelic loss of function in *DNAJC3*. This individual has a markedly different clinical course, with diazoxide-responsive hyperinsulinemic hypoglycemia persisting through adolescence as a dominant feature of the syndrome, marked short stature, and only subtle neurological abnormalities. The literature was reviewed for individuals with early-onset hypoglycemia and biallelic *DNAJC3* variants, with a specific focus on their metabolic profile. Additionally, young DNAJC3 knockout (K.O.) mice under 8 weeks of age, equivalence to human infancy, were studied in terms of beta-cell secretory function and glucose homeostasis. This provides potential mechanistic explanations for the biphasic phenotype.

## 2. Results

### 2.1. Clinical Report

The individual was born to non-consanguineous German parents via caesarean section at 39 weeks’ gestation due to breech presentation. The pregnancy was uneventful. The birth weight was 3410 g (+0.16 SD), length was 50 cm (−0.5 SD), and head circumference was 34.5 cm (−0.08 SD), all within normal range, and no dysmorphic features were observed. Further diagnostic evaluation was conducted within the first year of life due to severe growth retardation and moderate motor developmental delay. At 9 months of age, the patient was diagnosed with mild primary hypothyroidism (TSH 7.21 µU/mL, fT4 0.8 ng/mL) with negative thyroid autoantibodies, and L-thyroxine supplementation was initiated. A TRH stimulation test yielded normal results. By 13 months of age, the patient’s length was 65.5 cm (−4.0 SD) and body weight was 7.2 kg (−2.7 SD). The patient’s IGF-1 level and body length remained persistently low (<25 ng/mL and <−4 SD, respectively). Arginine infusion tests and insulin tolerance tests were used to exclude growth hormone deficiency at 13 months and 2.6 years of age. Insulin-like growth factor binding protein acid labile subunit (ALS), growth hormone binding protein (GHBP), IGF-1 secretion test, and a nocturnal profile of growth hormone secretion were found to be normal. Cystic fibrosis and celiac disease were ruled out. Imaging studies, including echocardiography, abdominal ultrasound, and cranial MRI, were unremarkable, except for two small, nonspecific, patchy hyperintense frontal white matter lesions. Karyotyping and CGH array revealed a normal female karyotype (46, XX). There were no mutations in the GH receptor gene, aldolase B (*ALDOB*), or short stature homeobox (*SHOX*). DNA methylation studies showed no abnormalities in the critical Prader-Willi region 15q11-q13.

At the age of 6 years, the child’s length was 97.9 cm (−4.2 SD), body weight was 16.7 kg (−2 SD), and IGF-1 levels were persistently low. A trial of off-label growth hormone treatment was initiated at a dose of 35 µg/kg/day. Although IGF-1 levels increased to the normal range, there was no growth response, and the treatment was discontinued after one year. Recombinant IGF-1 was administered at a dose of 250 µg/kg/day when the patient was 9 years old, but it had no effect on growth. The patient’s growth continued at a constant rate of around −4.0 SDS in the subsequent years. At the age of 14.8 years, the patient’s length was 144 cm (−3.8 SD) and body weight was 44.9 kg (−1.5 SD).

The patient experienced asymptomatic recurrent hypoglycemia (<40 mg/dL or 2.2 mmol/L) for the first time during hospitalization at 9 months of age due to rotavirus infection. Several tests were conducted to exclude disorders of glycogen storage, fatty acid oxidation, ketogenesis, and cortisol metabolism. The initial diagnosis of hypoglycemia was attributed to a feeding disorder with hypocaloric supply before further evaluation.

At the age of 2 years, she was referred to our center for further evaluation of hypoglycemia. During the formal fasting tolerance test, blood glucose concentrations remained below 70 mg/dL (<3.9 mmol/L) throughout the day and night. After 6 h of fasting, the glucose concentration reached a minimum of 44 mg/dL (2.4 mmol/L), followed by a spontaneous increase, but remained below 70 mg/dL (<3.9 mmol/L) until 20 h of fasting. During hypoglycemia below 50 mg/dL (<2.8 mmol/L), insulin levels were repeatedly moderately elevated between 2.7 and 3.7 mU/L. At the time of glucose nadir, betahydroxybutyrate (BHB) was low (0.1 mmol/L), but then progressively increased during continued fasting along with a spontaneous increase in blood glucose. During the patient’s hospital stay, BHB levels repeatedly remained low (≤0.1 mmol/L) during spontaneous hypoglycemia episodes. However, on several occasions, there was a delayed increase in response to hypoglycemia. Glucagon was effective in raising blood glucose levels by more than 30 mg/dL (1.7 mmol/L) during hypoglycemia. The patient’s plasma amino acids, urine organic acids, and plasma acylcarnitine profile were within the normal range, despite a moderate increase in 3-hydroxybutyrylcarnitine (C4OH). The sequencing of the hydroxyacyl-coenzyme A dehydrogenase (*HADH*) gene was normal, thus excluding short-chain 3-hydroxyacyl-coenzyme A dehydrogenase hyperinsulinism (SCHAD-HI).

The patient was diagnosed with CHI and treated with diazoxide at doses ranging from 7.5 to 10 mg/kg/day until the age of 8 years. Blood glucose concentrations remained stable between 60 and 80 mg/dL (3.3–5 mmol/L). The dosage of diazoxide was gradually reduced over time to 4 mg/kg/day by the age of 12 years. At the age of 14 years, diazoxide was paused for formal reevaluation of the metabolic profile. The patient exhibited persistent hyperinsulinemic, hypoketotic hypoglycemia during a standardized fasting test. After only 7 h, the blood glucose dropped to 52 mg/dL (2.9 mmol/L), while insulin was 6.5 mU/L, c-peptide was 1.4 ng/mL, free fatty acids (FFA) were 0.63 mmol/L, and BHB was 0.1 mmol/L. Throughout the fasting period, blood glucose levels stabilized between 55 and 69 mg/dL (3.1–3.9 mmol/L). However, after 20 h of fasting, the blood glucose concentration dropped again to 47 mg/dL (2.6 mmol/L) with elevated insulin levels and a subnormal metabolic fasting reaction (insulin 8.7 mU/L, c-peptide 1.48 ng/mL, FFA 1.0 mmol/L, BHB 0.4 mmol/L). The HbA1c level was 4.5%. Diazoxide is currently being administered at a dose of 2.2 mg/kg/day, resulting in blood glucose levels consistently above 70 mg/dL (3.9 mmol/L) throughout the day, including during a fasting period over more than 12 h. No formal fasting tolerance test was performed while under diazoxide treatment.

At the age of 7 years, sequencing was repeated and extended using targeted next-generation sequencing, which revealed no mutations in known CHI genes. However, two compound-heterozygous variants, c.83–2A>G and c.566delT (p.Trp186Glyfs*14), were found in *DNAJC3*. According to the ACMG criteria, both variants are considered as pathogenic. The first variant is predicted to result in the loss of an acceptor splice site, while the second variant causes a frameshift resulting in a premature stop codon that halts protein synthesis.

The individual experienced mild gross and fine motor developmental delay throughout infancy and adolescence, while cognitive development was normal. At the age of 7 years, bilateral high frequency sensorineural hearing loss (HF-SNHL) was diagnosed, requiring hearing aids. At 9.6 years of age, nerve conduction studies were consistent with mild sensorimotor demyelinating peripheral neuropathy, both of which are known features of a *DNAJC3* loss of function [11].

### 2.2. Metabolic Profile of Individuals with Congenital Hyperinsulinism Due to DNAJC3 Deficiency

Table 1 summarizes the metabolic profile of seven individuals from six families with biallelic *DNAJC3* mutations and early-onset hypoglycemia. Five of these individuals with CHI have recently been published (Individuals I3–I7). The series is supplemented with two additional individuals. One of them (Individual I1) was treated in our department and has not been previously reported. The other individual (Individual I2) was published by Synofzyk et al. and their chart review retrospectively identified early-onset hypoglycemia that had not been reported in the original article. Hypoglycemia was an incidental finding during the clinical workup of symptoms associated with DNAJC3 deficiency, particularly growth retardation, in all except one individual (Individual I3). However, Ozon et al. reported that two individuals had a history of afebrile convulsions, which led to the diagnosis of hypoglycemia in Individual 3, but remained uninvestigated in Individual 4 [6]. Most individuals were diagnosed with hypoglycemic episodes within the first year of life. Biochemical workup during hypoglycemia revealed hypoketotic hyperinsulinemic hypoglycemia in all individuals except for Individual 6, whose metabolic profile during hypoglycemia was not reported. Four individuals were treated with diazoxide, and all responded well. Three individuals required only frequent feeds with or without cornstarch. At ages 12 to 13.9 years, four individuals had already developed diabetes mellitus. At the time of publication or last metabolic assessment, three individuals were still receiving diazoxide treatment at ages 5, 10, and 14 years. Individual 6 and Individual 7, as reported by Ocansey et al., exhibited an age-appropriate fasting tolerance with decreased diazoxide requirements at ages 5 and 10 years, respectively [5].

### 2.3. Fasting Blood Glucose Concentrations, Glucose Tolerance and Plasma Insulin Concentrations in Young DNAJC3 K.O. Mice Compared to Wild-type Littermates

**Figure 1:**
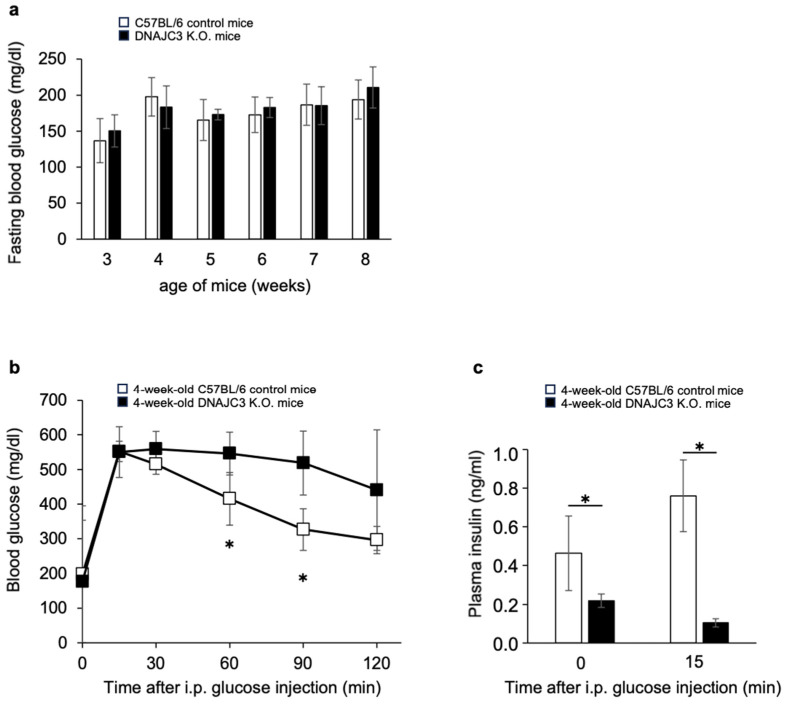
Fasting blood glucose concentrations, glucose tolerance, and plasma insulin concentrations in adolescent DNAJC3 knockout (K.O.) mice compared to C57BL/6 control mice. (**a**) Fasting blood glucose concentrations in 3–8-week-old C57BL/6 mice compared to DNAJC3 K.O. mice, fasted for 16 h once a week (*n* = 7 male mice per group). (**b**) Blood glucose concentrations of 4-week-old mice during an intraperitoneal glucose tolerance test (1.5 mg g^−1^ body weight); same mice as in a) (*n* = 6 DNAJC3 K.O. mice and *n* = 7 C57BL/6 mice). (**c**) Plasma insulin concentrations of 4-week-old mice during the intraperitoneal (i.p.) glucose tolerance test shown in (**b**). Statistical significance determined by an unpaired two-tailed Student’s *t*-test; all values are the mean ± SD. * *p* < 0.05.

To determine fasting blood glucose concentrations, 3–8-week-old DNAJC3 K.O. and C57BL/6 control mice were fasted once a week overnight for 16 h. From 3 to 8 weeks of age, there was no difference in fasting blood glucose concentrations between DNAJC3 K.O. and control mice (Figure 1a). However, while fasting blood glucose concentrations were similar in both groups, glucose tolerance was already significantly impaired (Figure 1b) and plasma insulin concentrations before and during the intraperitoneal glucose tolerance test were already significantly reduced in 4-week-old DNAJC3 K.O. mice (Figure 1c).

### 2.4. Islet Insulin Content and Insulin Release from DNAJC3 K.O. Islets Compared to Control Islets

**Figure 2:**
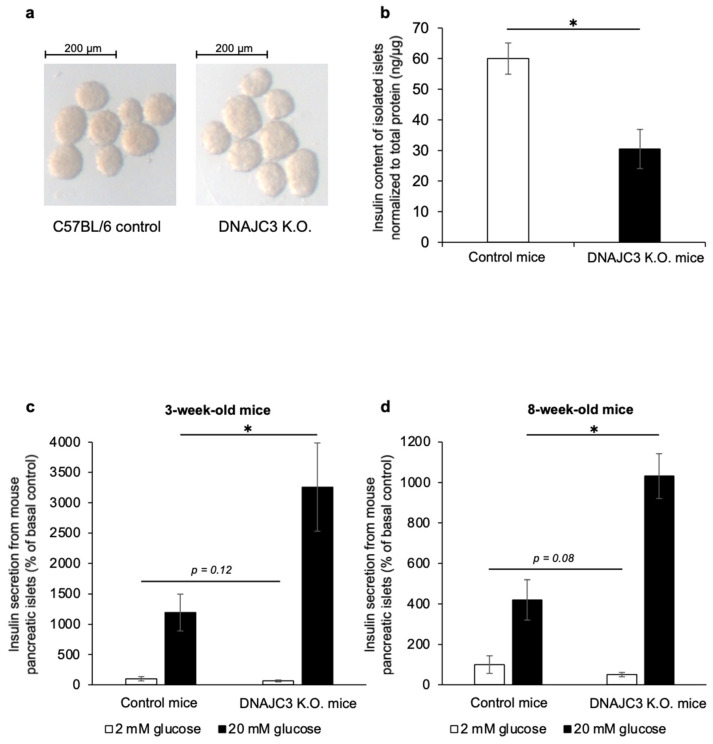
Insulin content and glucose-stimulated insulin secretion (GSIS) of control and DNAJC3 K.O. islets. (**a**) Representative light microscopy images of pancreatic islets from 4- to 6-week-old C57BL/6 control or DNAJC3 K.O. mice. (**b**) Islet insulin content of 3-week-old C57BL/6 control or DNAJC3 K.O. mice (*n* = 5 islet batches each. (**c**,**d**)) Insulin secretion from 3-week-old (**c**) and 8-week-old (**d**) control and DNAJC3 K.O. islets; *n* = 5 islet batches each. Statistical significance determined by an unpaired two-tailed Student’s *t*-test; all values are the mean ± SD. * *p* < 0.05.

Pancreatic islets that were isolated from 4- to 6-week-old DNAJC3 K.O. mice had a pale appearance when compared to control islets (Figure 2a). The insulin content of these islets was significantly lower than that of control islets (Figure 2b). Most importantly, while insulin release under low glucose conditions was similar in islets from 3- and 8-week-old DNAJC3 K.O. mice and control littermates, glucose-stimulated insulin release was significantly higher in islets that lacked DNAJC3 (Figure 2c,d).

## 3. Discussion

This report presents a case study of an individual with a biallelic loss of function of *DNAJC3* who has hyperinsulinemic hypoglycemia (HH) that persists into adolescence, but thus far only mild neurological manifestations. We also review the metabolic phenotype of published individuals with DNAJC3 deficiency and present in vivo and in vitro studies using a DNAJC3 knockout mouse model. Our findings suggest that uncontrolled calcium flux from the ER to the cytosol is the most likely mechanism triggering insulin release in DNAJC3 deficiency.

In 2014, biallelic *DNAJC3* mutations were first reported to cause a multisystem disorder including severe growth retardation, central and peripheral neurodegeneration, and juvenile-onset diabetes mellitus. Subsequent reports have expanded the phenotype to include hypothyroidism, retinal dystrophy, and congenital hyperinsulinism that progresses to diabetes mellitus in adolescence [5,6,7,8,9,10,11]. The clinical features of all reported individuals have been summarized previously [6,9]. It is noteworthy that in our individual (Individual 1), apart from hyperinsulinemic hypoglycemia and short stature, there were no other symptoms suggestive of *DNAJC3* deficiency in early childhood. It was not until the age of 7 years that sensorineural hearing loss and later on mild demyelinating peripheral neuropathy became apparent. Diazoxide was effective in treating hyperinsulinemic hypoglycemia, as reported by Ocansey et al. and Ozon et al. [5,6]. Despite being 14 years old and clinically mid-pubertal, the patient’s fasting tolerance remains significantly reduced, and she continues to require low-dose diazoxide treatment. There is no clear clinical evidence of progression of neurological disease.

Preclinical data on the role of DNAJC3 in maintaining ER homeostasis and, in particular, beta-cell survival are consistent with the development of diabetes mellitus in the absence of DNAJC3. *DNAJC3* is ubiquitously expressed in human tissues, including the pancreas, pancreatic islets, and liver [9,17]. Genetic deletion of *Dnajc3* is associated with beta-cell apoptosis in INS1-E cells, primary rat beta cells, and human islet cells in vitro [9]. In mice, *Dnajc3* knockout results in the gradual onset of hyperglycemia as a consequence of beta-cell apoptosis, reduced beta-cell mass, and hypoinsulinemia at adult maturity [15,16]. However, it remains unclear what causes HH, which subsequent reports have shown to be a typical feature of DNAJC3 deficiency in early childhood. Lytrivi et al. suggested periods of accumulated beta-cell death and insulin ‘leakage’ into the circulation as the underlying cause [9]. However, if hyperinsulinism was simply a consequence of beta-cell apoptosis, the increased insulin release should be short-lived, and those individuals most severely affected by HH would be expected to progress most rapidly to a state of insulin-deficient diabetes. As all the reported individuals had a low or normal BMI and none were obese, there was no evidence of insulin resistance contributing to ER stress and thus diabetes development. The individual presented here (Individual 1) was severely affected by HH with diazoxide requirements of up to 10 mg/kg/day. However, she has not yet developed diabetes mellitus at the age of 14 years. Moreover, the effectiveness of diazoxide in all described individuals raises questions about how it could reduce insulin secretion if it is mediated by beta-cell apoptosis.

A fatty liver phenotype, impaired gluconeogenesis, and depletion of hepatic glycogen stores have been proposed as alternative mechanisms for hypoglycemia in DNAJC3 deficiency [6]. Mice with genetic deletions of either ER stress-sensing pathways (*ATF6α, eIF2α, IRE1α*) or quality control (*Dnajc3*) show a similar response to ER stress, including the development of hepatic steatosis [18]. DNAJC3 K.O. mice develop inhibition of the transcriptional regulator C/ebpa, which regulates hepatic gluconeogenesis and lipogenesis [18]. Consistent with these findings, functional studies in fibroblasts from individuals with *DNAJC3* mutations revealed cellular lipid accumulation and increased sensitivity to cholesterol stress, leading to UPR activation, β-amyloid accumulation, and the impairment of mitochondrial oxidative phosphorylation [19]. Notably, mice deficient in the ER stress-sensor eIF2α die within hours of birth due to hypoglycemia associated with impaired gluconeogenesis [20]. However, the clinical data presented here argue against hepatic glycogen depletion and impaired gluconeogenesis as the major driving factor for hypoglycemia, as all individuals presented with hyperinsulinemic hypoglycemia, strongly suggesting insulin secretory dysfunction. However, at least in our patient, there were periods of elevated ketones during prolonged fasting or after hypoglycemia despite elevated insulin levels, which may be related to some degree of impaired gluconeogenesis.

We propose that the most likely mechanism for HH in DNAJC3 deficiency is uncontrolled calcium flux from the ER to the cytosol, triggering insulin release. The ER is a major storage compartment for calcium ions, with a free calcium concentration in the ER lumen of 100–800 µM, but a cytosolic free calcium concentration of only 50–100 nM in resting mammalian cells. The calcium gradient between the ER lumen and the cytosol is tightly regulated by a variety of calcium handling enzymes, proteins, channels, and transporters that work together to provide a temporally and spatially precise calcium signal. This allows calcium to play a central role as a second messenger in cellular signaling [21,22]. Passive calcium efflux (calcium leakage) from the ER into the cytosol is a major component that influences intracellular calcium homeostasis. Previous research has identified the Sec61 polypeptide-conducting channel as a calcium leakage channel. The Sec61 complex facilitates signal-peptide-dependent protein transport across the ER membrane. Once protein translocation is complete, the Sec61 complex allows calcium efflux from the ER into the cytosol along the concentration gradient [22,23,24]. In HeLa cells, the ER luminal BiP protein restricts calcium leakage through the Sec61 channel by facilitating channel closure. The absence of available BiP leads to increased calcium leakage via the Sec61 complex [22]. Furthermore, genetic deletion of the ER-luminal BiP co-chaperones *Dnajc3* and *DnaJb11* results in increased calcium leakage through the Sec61 complex. Therefore, DNAJC3-controlled gating of Sec61 by BiP limits ER calcium leakage from the ER [25]. Beta cells are highly active in protein synthesis and secretion. Therefore, DNAJC3 deficiency is expected to cause increased calcium flux from the ER into the cytosol, triggering uncontrolled insulin release and thus HH in the early phase of the disease. However, if the demand for insulin synthesis and protein folding exceeds the capacity of the ER, and the unfolded protein response is unable to resolve the protein-folding defect due to the absence of DNAJC3, apoptosis is initiated. This leads to progressive beta-cell loss and the onset of diabetes mellitus during later stages of the disease, as demonstrated in DNAJC3-deficient mice.

In INS-1E cells, surprisingly, *Dnajc3* knockdown did not affect insulin content or glucose-stimulated insulin secretion [9]. Therefore, we studied young DNAJC3 K.O. mice, less than 8 weeks of age, equivalent to human infancy, to investigate insulin release, glucose homeostasis, and evidence of hypoglycemia. Our findings provide evidence that insulin release is indeed disturbed in islets isolated from 3- to 8-week-old DNAJC3 K.O. mice. Under low-glucose conditions, there was no difference. However, during periods of increased synthetic demand (high-glucose stimulation), K.O. islets showed significantly higher insulin release compared to the control. Additionally, islet insulin content was already significantly lower in DNAJC3-deficient islets from 3-week-old mice compared to age-matched controls. In vivo, episodes of hypoglycemia were not detected in DNAJC3-deficient mice during either fasting or glucose challenge. During an intraperitoneal glucose tolerance test, basal and stimulated plasma insulin concentrations were found to be decreased in 4-week-old DNAJC3-deficient mice, and glucose tolerance was impaired.

We conclude that at the youngest experimentally accessible age of 4 weeks, the in vivo glycemic phenotype is already dominated by advanced ER stress-induced beta-cell apoptosis and reduced total insulin secretion capacity. However, at the cellular level, glucose-stimulated insulin release is increased during periods of increased protein synthesis and insulin demand conclusive with impaired Sec61 channel gating. 

In humans, however, there appears to be a distinct biphasic phenotype with increased insulin release and HH, which is believed to be caused by calcium leakage from the ER. This occurs before beta-cell apoptosis progresses to a state where the remaining beta-cell mass becomes the limiting factor for glycemic control. This genetic mechanism is noteworthy as it explains CHI solely through the disruption of intracellular calcium homeostasis. In contrast, previously known CHI genes all relate to the influx of extracellular calcium via voltage-gated calcium channels [3]. Future calcium imaging studies may provide additional evidence to support this hypothesis by examining intracellular calcium flux in DNAJC3-deficient islets.

## 4. Material and Methods

### 4.1. Index Patient

The University Children’s Hospital Duesseldorf in Germany identified the index patient and conducted clinical management and examinations. Genetic testing was performed as part of a clinical study (German Clinical Trials Register ID DRKS00006874), which was approved by the Ethics Committee of the Medical Faculty of the Heinrich Heine University Duesseldorf (ID 4790R). The study obtained written informed consent for inclusion and publication.

### 4.2. Genetic Studies

In brief, we utilized a customized sequence capture library that targeted the exons and additional 35 bp of flanking intronic sequences of the genes known or hypothesized to cause monosymptomatic or syndromic CHI or other forms of monogenic glucose dysregulation. Genomic DNA was fragmented and enriched using the Roche/NimbleGen sequence capture approach (NimbleGen, Madison, WI, USA), amplified, and sequenced simultaneously with Illumina NGS sequencing-by-synthesis technology using an Illumina HiSeq 1500 system (Illumina, Inc., San Diego, CA, USA). The mean target coverage was 624× on the HiSeq system, with about 99% of the target regions covered with at least 20×. NGS data analysis was performed using bioinformatic analysis tools, as well as the JSI Medical Systems software version 4.1.2 (JSI Medical Systems GmbH, Ettenheim, Germany). Further details on sequencing and bioinformatic analysis have been described elsewhere [26].

### 4.3. Mouse Model

Heterozygous C57BL/6-Dnajc3^tm8663Wcl^/Mmmh mice (*P58^IPK-^*) were obtained from the Mutant Mouse Resource and Research Centers (MMRRC, Columbia, MO, USA) after cryo-resuscitation by embryo transfer. The original strain was obtained from Warren C. Ladiges, Department of Comparative Medicine, University of Washington, USA. The generation of P58IPK-deficient embryonic stem cells and mice has been described previously [16]. The MMRRC provided heterozygous mice which were intercrossed to obtain DNAJC3 K.O. mice and C57BL/6-wild-type littermates (control mice) for experiments. The mice were housed in rooms with a controlled temperature of 22 °C, 55% humidity, and a 12:12 h light/dark cycle. They had ad libitum access to standard laboratory chow and drinking water. The animal experiments were approved by the local animal ethics committee of the Landesamt für Natur, Umwelt und Verbraucherschutz Nordrhein-Westfalen (LANUV North Rhine-Westphalia, Recklinghausen, Germany) under AZ 81-02.04.2018.A346. Routine genotyping was carried out using PCR and the following primers: P58-del-5′: AGC CCG GCC TCC CCA GCC TCT TC and P58-del-3′: CCC GTC CAC TCG CTC GCT CGC TC, with the addition of 0.05% DMSO and GoTaq G2 Hot Start Green Master Mix.

### 4.4. Isolation of Mouse Pancreatic Islets

Pancreatic islets from 3- to 4-week-old DNAJC3 K.O. and C57BL/6 wild-type littermates (control mice) were isolated, following the method described by Yesil et al. with minor modifications [27]. The pancreatic tissues were enzymatically digested with Liberase TL Research Grade (Roche, Basel, Switzerland) at 37 °C for 16 minutes. Digestion was stopped with DMEM and GlutaMAX (1 mg/mL glucose), supplemented with 15% heat-inactivated FBS (all Thermo Fisher Scientific, Waltham, MA, USA). Following several washing and filtering steps, islets were isolated from exocrine tissue through gradient centrifugation at 1200× *g* for 25 minutes. The islets were then collected from the interphase between Histopaque-1077 and DMEM. After being washed twice with islet medium (Connaught Medical Research Laboratories medium 1066, CMRL) supplemented with 15% heat-inactivated FBS, 100 U/mL penicillin, 100 µg/mL streptomycin, 50 µM β-mercaptoethanol, 0.15% NaHCO_3_, and 10 mM glucose, they were ready for further use. All additional assays were conducted following an overnight culture in islet medium at 37 °C, 5% CO_2_, and a humidified atmosphere.

### 4.5. Insulin Secretion from Isolated Pancreatic Islets and Measurement of Insulin Content

Pancreatic islets of similar size were isolated from DNAJC3 K.O. or C57BL/6 wild-type littermates (control mice) and starved for 1 hour in Krebs Ringer HEPES (KRH) buffer. The buffer contained 15 mM HEPES, 5 mM KCl, 120 mM NaCl, 2 mM CaCl_2_, 10 μM glycine, 24 mM NaHCO_3_, and 1 mg mL^−1^ bovine serum albumin, supplemented with 2 mM glucose. To determine insulin secretion under basal and glucose-stimulated conditions, we measured the amount of insulin secreted from the same islets at 2 mM glucose (low glucose) and 20 mM glucose (high glucose). The islets were incubated in fresh low-glucose KRH buffer for 1 h, followed by incubation in high-glucose KRH buffer for 1 h. The supernatants were collected to determine the amount of insulin secreted. We then lysed the islets in RIPA buffer to measure insulin content. Insulin secretion and content were measured using an ultrasensitive rat insulin ELISA (Crystal Chem, Zaandam, The Netherlands) in combination with an Infinite M200 NanoQuant reader (Tecan, Männedorf, Switzerland). Insulin secretion was normalized to total insulin and expressed as a percentage of basal control insulin secretion. To measure the insulin content, the insulin was normalized to the total protein content, which was measured using the bicinchoninic acid (BCA) method (Thermo Fisher Scientific, Waltham, MA, USA).

### 4.6. Fasting Test and Glucose Tolerance Test

To measure fasting blood glucose concentrations, we fasted 3-8-week-old DNAJC3 K.O. and C57BL/6 wild-type littermates (control mice) once a week for 16 h. We measured glucose concentrations twice in blood collected from the tail using the Monometer Futura glucometer (MedNet GmbH, Münster, Germany). For the glucose tolerance test, we injected 1.5 mg glucose/g body weight intraperitoneally (i.p.). Glucose concentrations were measured twice in blood collected from the tail before and at 15, 30, 60, and 120 min after i.p. injection. Insulin concentrations were measured before and 15 minutes after i.p. glucose injection using an ultra-sensitive rat insulin ELISA (Crystal Chem, Zaandam, The Netherlands) in combination with the GloMax Discover Microplate Reader (Promega, Madison, WI, USA).

### 4.7. Statistical Analysis

Statistical significance was determined as described in the figure legends. Calculations were performed using Excel version 16.77.1 (Microsoft Corporation, Redmond, WA, USA). The unpaired Student’s *t*-test was used to compare two groups, with *p* values < 0.05 considered significant. The data are presented as the mean ± standard deviation.

## 5. Conclusions

In summary, seven individuals with a biallelic loss of function of *DNAJC3* have now been reported to have early-onset hypoglycemia, most of whom were asymptomatic. In those investigated, a metabolic workup revealed HH. Thus, a biphasic endocrine phenotype appears to be a common finding in biallelic *DNAJC3* deficiency. It begins with congenital hyperinsulinism, resolves over time, and ultimately leads to diabetes, depending on the individual progression of beta-cell apoptosis. However, since hypoglycemia was often reported to be asymptomatic, the initial hyperinsulinemic phase may go unnoticed. Clinicians should therefore screen for HH in all individuals with biallelic *DNAJC3* mutations to avoid missing the initial phase. Conversely, when considering a differential diagnosis of CHI, DNAJC3 deficiency should be taken into account. Therefore, sequencing strategies for CHI should include *DNAJC3*. Other symptoms suggestive of DNAJC3 deficiency may be subtle in the early phases of the disease. The driving factor causing hypoglycemia in DNAJC3 deficiency appears to be increased islet insulin secretion, possibly caused by uncontrolled calcium leakage from the ER via the Sec61 complex, rather than insulin leakage from dying beta cells. Treatment with the chaperone 4-phenylbutyric acid (4-PBA), which is known to relieve ER stress, has been shown to preserve beta-cell mass and function in DNAJC3 K.O. mice [15]. Sodium phenylbutyrate, the organic sodium salt of 4-PBA, is approved for the chronic treatment of hyperammonemia due to urea cycle disorders in children and adults and is available as an oral solution. Further clinical studies should be conducted to determine whether PBA preserves beta-cell function in humans and thus delays the onset of diabetes mellitus in DNAJC3-deficient individuals. Additionally, it should be investigated whether PBA protects neurons to ameliorate progressive neurodegeneration.

## Figures and Tables

**Table 1:** Metabolic profile during hypoglycemia in individuals with congenital hyperinsulinism due to DNAJC3 deficiency.

**Baseline Characteristics**											
**Study**	present			Synofzik et al. *(personal communication)* [11]	Ozon et al. [6]				Lytrivi et al. [9]	Ocansey et al. [5]	
**individual**	*individual 1*			*Individual 2* *(Case II1 of Family 2 in Synofzik et al.)*	*Individual 3* *(Case 1 in Ozon et al.)*			*Individual 4* *(Case 2 in Ozon et al.)*	*Individual 5* *(Case 1 in Lytrivi et al.)*	*Individual 6* *(Case 1 in Ocansey et al.)*	*Individual 7* *(Case 2 in Ocansey et al.)*
**country of origin/migrational background**	Germany			Turkey	Turkey			Turkey	France/Armenia	UK/Pakistan	UK/Pakistan
**consanguinity**	no			yes	yes			yes	no	yes	yes
**sex**	female			female	male			female	female	male	male
**GA, BW (g), BL (cm)**	39+0; 3410; 50			37+x; 2930; 49	at term; 3750; NR			at term; 3100; NR	37 + 0; 2430; 46	41; 3300 g; NR	36; 2060; NR
**DNAJC3 mutation**	c.83-2A>G/c.566delT, p.Trp186Glyfs*14 (comp het)			deletion of exons 6–12 (p.?) (hom)	c.393+2 T>G, NM_006260.4 splice site mutation (hom)			c.393+2 T>C, NM_006260.4 splice site mutation (hom)	c.1036C>T, p.R346*/c.1A>G, p.M1V (comp het)	c.1367_1370delAGAA p.Lys456SerfsTer85 (hom)	c.1367_1370delAGAA p.Lys456SerfsTer85 (hom)
**Hypoglycemia**											
age at first documented hypoglycemia	9 month			3 years	2 years			5 years †	9 months	13 months	neonate
symptoms of hypoglycemia	asymptomatic			asymptomatic	convulsions			Asymptomatic †	NR	asymptomatic	asymptomatic
management of hypoglycemia	DZX (7.5–10 mg/kg/d)			frequent feeds/gastric tube	frequent feeds and cornstarch at night			frequent feeds, cornstarch, DZX	NR	DZX	DZX
**metabolic profile during hypoglycemia**											
age at formal testing/diagnosis	2 y	3.5 y	14 y	3 y	2 y	6.5 y		5–8.3 y	NR	NR	2.8 y
glucose (mmol/L) [mg/dL]	2.5 [46]	3.1 [56]	40	2.2 [40]	1.78 [32]	2.9 [52]	2.6 [47]—2.4 [43] ^§^	2.6 [47]—2.3 [41]	NR	38 (2.1)	45 (2.5)
insulin (mU/L)	3.2	6.4	6.5	3.93	<2.0	6	4.3–6.9 ^§^	3.4–9.3	NR	3.8	4.6
c-peptide (ng/mL)	1.32	NA	1.4	NR	NR	0.51	NR	NR	NR	NR	NR
FFA (mmol/L)	NA	NA	0.63	NR	NR			NR	NR	0.35	0.3
BHB (mmol/L)	NA	0.1	0.1	NR	NR			NR	NR	<0.05	<0.05
urinay ketones	NA	NA	NA	NR	negative	negative	negative	negative	NR	negative	negative
fasting tolerance at last evaluation (age)	7 h (14 y)			NR	16 h (6.5 y)			NR	NR	age appropriate	age appropriate
**Diabetes mellitus (DM)**											
age at diagnosis (y)	no DM age 14 y			13.6	12.5			13.9	12	no DM age 10 y	no DM age 5 y
HbA1c (%)	4.5			11.1	7.1			5.9	NA	NR	NR
management of hyperglycemia	---			insulin treatment	insulin treatment			diet and exercise	OAD; GLP-1 RA since age 26 y	---	---

Abbreviations: BL: body length; BW: body weight; DZX: diazoxide; FFA: free fatty acids; BHB: betahydroxybutyrate; GA: gestational age; GLP-1 RA: glucagon-like peptide-1 receptor agonist; NA: not available, NR: not reported, OAD: oral antidiabetic drug. ^§^ hypoglycemia induced by levodopa; ^†^ history of afebrile seizure at 2.5 y which was uninvestigated.

## Data Availability

The datasets are available on request.
